# Deterministic reshaping of single-photon spectra using cross-phase modulation

**DOI:** 10.1126/sciadv.1501223

**Published:** 2016-03-25

**Authors:** Nobuyuki Matsuda

**Affiliations:** NTT Basic Research Laboratories, NTT Corporation, Atsugi, Kanagawa 243-0198, Japan. E-mail: m.nobuyuki@lab.ntt.co.jp

**Keywords:** nonlinear optics, quantum optics, optical fibers, cross phase modulation, Single photons, Quantum information processing, quantum entanglement.

## Abstract

The frequency conversion of light has proved to be a crucial technology for communication, spectroscopy, imaging, and signal processing. In the quantum regime, it also offers great potential for realizing quantum networks incorporating disparate physical systems and quantum-enhanced information processing over a large computational space. The frequency conversion of quantum light, such as single photons, has been extensively investigated for the last two decades using all-optical frequency mixing, with the ultimate goal of realizing lossless and noiseless conversion. I demonstrate another route to this target using frequency conversion induced by cross-phase modulation in a dispersion-managed photonic crystal fiber. Owing to the deterministic and all-optical nature of the process, the lossless and low-noise spectral reshaping of a single-photon wave packet in the telecommunication band has been readily achieved with a modulation bandwidth as large as 0.4 THz. I further demonstrate that the scheme is applicable to manipulations of a nonclassical frequency correlation, wave packet interference, and entanglement between two photons. This approach presents a new coherent frequency interface for photons for quantum information processing.

## Teaser

With an ultrafast refractive index change in an optical fiber, frequency entanglement of twin photons is modulated on the fly.

## INTRODUCTION

The photon appears to be a natural choice as an information carrier in quantum communication, which facilitates entanglement distribution over long distances. Such quantum networking (*1*), which offers opportunities for various technologies in the field of quantum information processing (*2*–*4*), requires a coherent optical link to be established between physical systems that have disparate properties such as frequency responses. Thus, the capacity to harness the center frequency and shape of a photon spectrum is vital (*5*). Such technology will also provide direct control of the quantum state of photons encoded in a frequency degree of freedom of light, which naturally serves a wealth of state space for large-scale quantum computation and simulation (*6*–*10*).

Since its first experimental demonstration in 1992 (*11*), the center frequency conversion of nonclassical light has been widely investigated using nonlinear three- or four-wave mixing (FWM) (*12*–*19*), where photon frequencies can be converted with mediation provided by the energy of other input pump fields. The intense pump required for highly efficient conversion tends to simultaneously create noise photons, whereas the demand for scalable quantum networking provides strong motivation for research into the simultaneous realization of lossless and noiseless conversion; for example, an internal efficiency of more than 80% with a signal-to-noise ratio of over 100 was demonstrated for a single-photon state (*18*). Furthermore, the other key function, spectral reshaping, was recently appended to the frequency conversion processes (*20*–*22*), and research aimed at high-efficiency reshaping of single photons is in progress (*23*, *24*). At the same time, the search for other schemes that can harness the spectral property of photons, ideally based on a deterministic process, may offer a new approach toward scalable quantum networking.

Another nonlinear optical interaction that enables frequency conversion is cross-phase modulation (XPM) (*25*, *26*), which is a third-order nonlinear optical effect that enables the phase of an optical field to be controlled by another field. XPM provides a function for spectral reshaping via dynamic phase alteration of an optical field, which is widely applied to the frequency conversion of high-speed optical signals (*26*), to biological imaging (*27*), and even to testing the event horizon (*28*) in the classical regime. The XPM-induced phase shift at the quantum level has been extensively investigated (*29*, *30*) with a view to realizing scalable quantum logic gates (*4*) and quantum nondemolition measurement (*31*), thanks to the deterministic nature of the XPM interaction (*32*). Thus, it also has the potential for realizing highly efficient spectral reshaping that is applicable to single photons. Experiments in the classical regime achieved reshaping with a conversion bandwidth of more than 1 THz (*26*) as well as key tasks for communication including uniform spectral shifting (*33*), spectral compression (*34*), time lensing (*35*), and pulse retiming (*36*). However, XPM-based spectral manipulation has not yet been applied to single photons.

## RESULTS

Here, I present the first experimental reshaping of spectral distributions of single photons using XPM. The deterministic nature of the process made it possible to realize the spectral conversion of single-photon wave packets without an observable interaction loss caused by the conversion. Using the high bandwidth of the dynamic frequency shift induced by subpicosecond optical pulses, I report the postgeneration conversion of the nonclassical joint spectral correlation of photon pairs. Furthermore, I show that the wave packet interference and entanglement between the reshaped single photon and another photon remain, directly demonstrating the applicability of XPM reshaping to quantum information technology.

### Experimental method

**Fig. 1:**
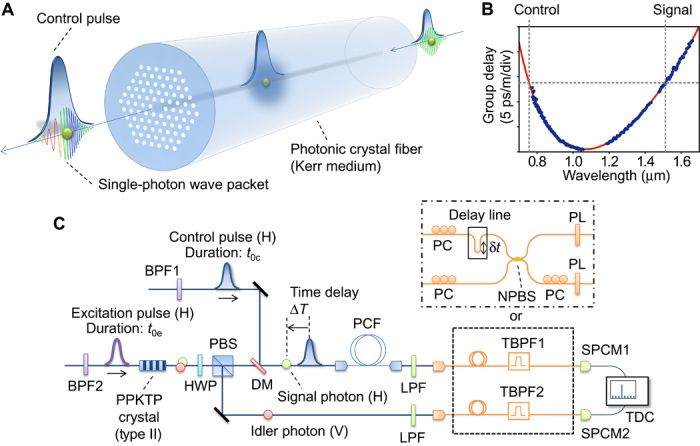
Spectral reshaping of a single-photon wave packet using XPM. (**A**) Conceptual illustration of the scheme. The control pulse (displayed as an envelope) induces a dynamic nonlinear phase shift, that is, an instantaneous frequency modulation of the single-photon wave packet via XPM in the Kerr medium. (**B**) Measured group-delay spectrum of the photonic crystal fiber (PCF) used. The solid curve represents a sixth-order polynomial fitting. (**C**) Schematic of the experimental setup. The control pulse for XPM and the excitation pulse for photon pair generation are obtained from a pulsed laser source. PBS, polarizing beam splitter; DM, dichroic mirror; LPF, long-pass filter; (T)BPF, (wavelength-tunable) band-pass filter; PL, polarizer; PC, fiber polarization controller; NPBS, nonpolarizing 50/50 fiber beam splitter; SPCM, single-photon counting module; TDC, time-to-digital converter. H and V represent horizontal and vertical polarizations, respectively. The paths of signal and idler photons are swapped by the half-wave plate (HWP) in the experiment for entanglement detection (Fig. 4, D to F).

A wave packet consisting of a single photon acquires a new frequency component via XPM induced by an intense control pulse propagating at the same speed in an optical fiber as in an optical Kerr medium (Fig. 1). In the classical picture, the presence of a control pulse with an intensity profile *P*(*t*) leads to an intensity-dependent variation in the refractive index of the material, which is experienced by the signal field as a phase shift φ(*t*) ∝ *P*(*t*). This gives rise to the instantaneous frequency shift Δω(*t*) = −*d*φ(*t*)/*dt* (*25*). Unlike frequency mixings, spectral reshaping is not accompanied by the center frequency translation of the modulated photons.

The dynamic frequency shift of single photons has also been demonstrated using electro-optic (EO) modulators driven by a radio-frequency electric field (*7*, *37*, *38*). Nonetheless, XPM can provide a larger conversion bandwidth [>1 THz (*26*)] than that obtained with the EO modulator approach because the control field is an optical pulse that can be made ultrafast in the femtosecond regime. This is useful for manipulating the frequencies of photons generated in a spontaneous process in nonlinear crystals, which have played pivotal roles in a number of quantum information experiments (*39*–*43*). Furthermore, Δω(*t*) can be controlled simply by tailoring the control pulse envelope function *P*(*t*). Hence, the all-optical scheme will offer various spectral reshaping capabilities (*33*–*36*) incorporated with well-developed ultrafast pulse shaping technology (*44*).

The quantum field to be converted now has an intensity that is considerably lower than that of the control pulses. Accordingly, a large frequency separation is needed between the two interacting fields to eliminate potential noise caused by the nonlinear spectral broadening of the control pulse. Moreover, the two interacting fields must propagate at the same pace; otherwise, φ(*t*) will become constant over the entire wave packet and cancel the frequency shift.

Photonic crystal fiber (PCF) (*45*) is a Kerr medium that fulfills this criterion. I used a PCF (NL-5.0-1065 from NKT Photonics A/S, 1 m long) that has the dispersion property shown in Fig. 1B, which I measured using white light cross-correlation interferometry (*46*). It exhibits two widely separated wavelengths with the same group velocity, thanks to the dispersion property, which is managed by arranging the air-hole claddings that surround the silica core (*45*). Such a dispersion engineering property, unachievable by standard optical fibers, is widely used in various applications including supercontinuum generation (*45*), FWM-based quantum frequency conversion (*15*), and test beds for single-photon nonlinearity (*30*) and the event horizon (*28*).

Figure 1C shows a schematic illustration of the experimental setup. Here, I reshape the frequency of one of the correlated photons (labeled signal and idler) created via type II spontaneous parametric down-conversion (SPDC) in a periodically poled potassium titanyl phosphate (PPKTP) crystal (*41*). The center wavelength of the control field λ_c_ was chosen to be 756 nm, which satisfies *v*_g_(λ_c_) ~ *v*_g_(2λ_c_), where *v*_g_ is the group velocity of the PCF. In this way, both the XPM control pulses and SPDC excitation pulses can be fed from a pulsed laser source (see Materials and Methods for details).

### Reshaping spectral distribution of photons

**Fig. 2:**
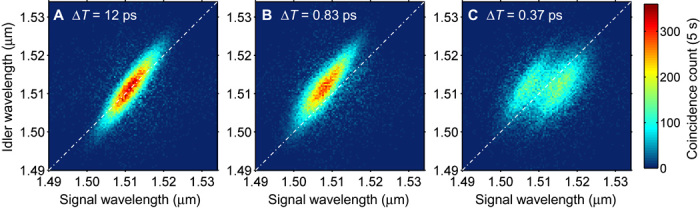
Reshaping biphoton frequency correlations. (**A** to **C**) The time delay between the signal photons and the control pulses, Δ*T*, was set so that the signal photon spectrum was (A) unchanged (original JSA), (B) blue-shifted, and (C) broadened. The zero-detuning lines ω_s_ = ω_i_ are represented by the dot-dashed lines as a reference.

In Fig. 2, I demonstrate the reshaping of the frequency correlation by plotting the measured joint spectral intensity (JSI) |*S*(ω_s_,ω_i_)|^2^ of the photon pairs for various time delays, Δ*T*, of the signal photon with respect to the control pulses. Here, ω_s(i)_ is the angular frequency of the signal (idler) photons and *S*(ω_s_,ω_i_) is the joint spectral amplitude (JSA) of a biphoton whose state is described by $|\psi \rangle \propto \int d\omega_{s}d\omega_{i}S(\omega_{s},\omega_{i}){\hat{a}}_{s}^{†}(\omega_{s}){\hat{a}}_{i}^{†}(\omega_{i})|0\rangle$, where ${\hat{a}}_{s(i)}^{†}(\omega_{s(i)})$ is the creation operator of a photon in the signal (idler) mode (*40*). Because there is no XPM for Δ*T* values much larger than the temporal width of the control pulses (0.78 ps), the JSI in Fig. 2A is identical to the initial JSI of the photon pairs emitted from the source (see fig. S1). At Δ*T* = 0.83 ps, where the signal photons are mainly synchronous at the trailing edge of the control pulses in the PCF, the JSI is entirely blue-shifted along the axis of the signal wavelength (Fig. 2B). Here, the frequency shift is approximately 3.2 nm (0.4 THz). This shows that the frequency correlation between photons is transferred to new frequency sets. When the photons stay at around the peak position of the control pulse in the PCF (Δ*T* = 0.37 ps, Fig. 2C), the JSI is broadened along the horizontal axis, revealing a further reshaping capability. Control of the JSI (JSA) is an essential task in quantum information science and has been developed by engineering the characteristics of nonlinear crystals or pump pulses for photon pair generation (*40*, *47*). The result constitutes the first experimental observation of postgeneration conversion over the JSI via a frequency conversion scheme.

**Fig. 3:**
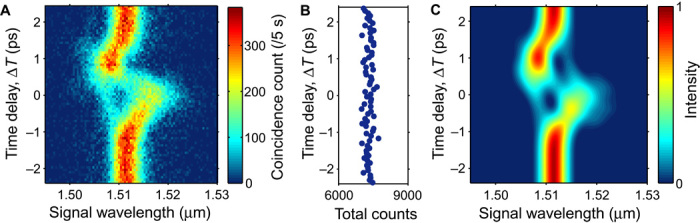
Delay dependence of the marginal spectrum of heralded signal photons. (**A**) Experimental result. Coincidence counts were recorded as a function of the center wavelength of TBPF1, whereas that of TBPF2 was fixed at 1512 nm. (**B**) Sum of the coincidence counts for each time delay. The total coincidence count is unchanged regardless of the XPM interaction. (**C**) Numerical simulation result based on nonlinear-coupled Schrödinger equations (see section SI for details).

Figure 3A shows the Δ*T* dependence of the signal photon spectra heralded by the detection of idler photons. Here, the center wavelength of the band-pass filter for the idler photons [tunable band-pass filter 2 (TBPF2)] is fixed at 2λ_c_ (1512 nm) to make it possible to observe the signal photon component that was originally correlated with the idler photons at that wavelength. The heralded spectrum is markedly modified as Δ*T* varies. For instance, as expected (*25*), most of the spectrum is red- or blue-shifted when photons are at the trailing or rising edge of the control pulses, respectively. Figure 3B is a plot of the sum of the coincidence counts for each Δ*T*. The total coincidence count remains constant regardless of the delay, demonstrating that the conversion successfully occurred without an observable photon loss at least for a data fluctuation of 2.2% (SD). A numerical simulation of the frequency modulation is performed on the basis of coupled nonlinear Schrödinger equations including XPM, self-phase modulation, and dispersion (see section SI). The simulation result is obtained, as shown in Fig. 3C, with reasonable free-fitting parameters that describe well the characteristics of the spectral shifts seen in the experimental data.

### Control over nonclassical interference between photons

**Fig. 4:**
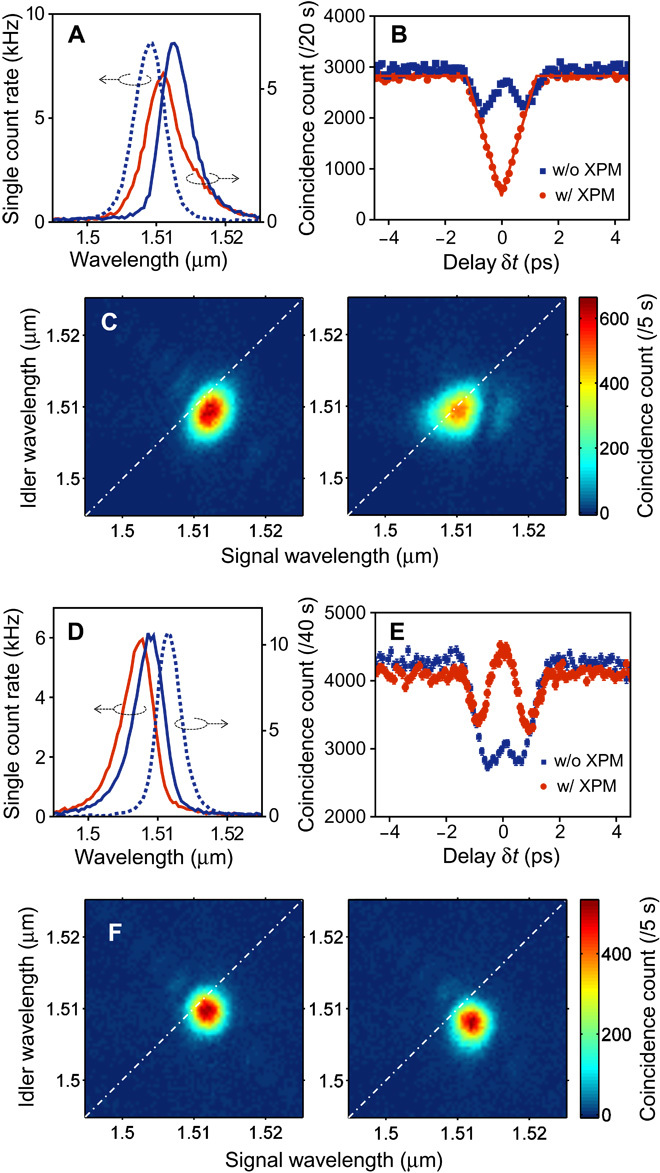
Control over nonclassical interference between photons. (**A** to **C**) Engineering biphoton distinguishability in frequency. (A) Single count spectra of signal photons without (blue solid curve) and with (red solid curve) XPM and idler photons (blue dashed curve). (B) Two-photon interference fringes without (blue) and with (red) XPM reshaping. (C) Two-photon JSI without (left) and with (right) XPM. (**D** to **F**) Detection of two-photon frequency entanglement after the application of XPM. (D) Single count spectra of signal photons (blue dashed curve) and idler photons without (blue solid curve) and with (red solid curve) XPM. (E) Two-photon interference fringes without (blue) and with (red) XPM, each with different axes for comparison. The bump with XPM reshaping indicates that the entangled component in JSA acquired an antisymmetric wave function. (F) JSI without (left) and with (right) XPM. In (C) and (F), the zero-detuning lines are provided by the dot-dashed lines as a reference.

Next, I use the reshaping scheme to tailor a two-photon interference, which lies at the heart of photonic quantum information technologies (*1*–*4*). The observation of this two-photon interference with frequency-converted photons is also a preliminary step toward the construction of a quantum network using distinct physical systems and quantum information processing using photons with different colors (*13*, *18*). In the following, the interference is shown to occur even after XPM reshaping. The experimental setup is modified (see Materials and Methods) so that the Hong-Ou-Mandel (HOM) interference (*39*) will occur at the NPBS if the signal and idler photons are indistinguishable as regards any physical degree of freedom. I start with photon pairs with nondegenerate center wavelengths whose single-count spectra and JSI are shown in Fig. 4A (blue curves) and Fig. 4C (left). For these photons, a two-photon interference fringe, which is the coincidence count rate *R* versus the signal-to-idler arrival time difference δτ at NPBS (provided by the delay line), is observed as shown in Fig. 4B (blue squares). The poor visibility is due to the small spectral overlap (23%) between the initial photons.

Then, an XPM-induced blue shift is applied to the signal photons. When Δ*T* = 0.88 ps, the spectrum of the signal photons is altered into the distribution shown by the red curve in Fig. 4A, which attained an increased spectral overlap of 70% with that of the idler photons. Now, I obtain an interference fringe exhibiting a clear HOM dip displayed as red dots in Fig. 4B. The visibility of the dip, *V* = |(*R*_classical_ − *R*_quantum_)/*R*_classical_|, is 0.84 ± 0.01. This exceeds the classical limit of 0.5 and confirms that the coalescence of the photon pairs took place at the NPBS thanks to the successful reduction of biphoton distinguishability. Without the accidental (background) coincidence count, *V* = 0.87 ± 0.01, which is explained by the upper-bound visibility of 0.91 calculated (see section SII) from the JSI after XPM (Fig. 4C, right). The triangular shape of the dip is Fourier-related to the sinc-shaped JSA (see section SI) of the correlated photons created in SPDC (*38*, *40*), as the small side lobes of the sinc squared function of the JSI can be slightly seen in the measured data. The low background levels in Fig. 4A, from which only detector dark counts have been subtracted, demonstrate that reshaping is accompanied by low noise. The observed background single count induced by the control field is less than 100 Hz per TBPF window (0.4 nm). *R*_classical_ (*R* at a sufficiently large δτ) is largely maintained regardless of XPM thanks to the lossless conversion. The subtle discrepancy is due to the nonlinear polarization rotation of the signal photons (see section SIII for details).

### Manipulation of biphoton entanglement in frequency

In a two-photon interference setup, a bump will appear in the coincidence count when biphotons have an antisymmetric (fermionic) wave function in a subspace, such that *S*(ω_s_,ω_i_) = −*S*(ω_i_,ω_s_) in frequency, where the symmetry refers to the exchange of particles (*42*, *48*). This is because the entire photon (boson) wave function is inherently symmetric, and thus an antisymmetric wave function introduces antisymmetry into the spatial part of the wave function, which is filtered by the NPBS. This results in antibunching of the photons, which leads to an *R* greater than *R*_classical_, the observation of which is a sufficient criterion for entanglement (*42*, *48*). Such phenomena are also observed by introducing an antisymmetric wave function in other subspaces such as polarization (*43*). Now, I attempt to observe the phenomenon by increasing the antisymmetric part in a frequency subspace via XPM.

Starting with the photon pairs whose spectra are shown as blue curves in Fig. 4D and the left panel in Fig. 4F, I obtain the two-photon interference fringe shown as blue squares in Fig. 4E. Then, the marginal idler spectrum is reshaped so that the JSA of the photon pairs will gradually acquire antisymmetric components. When the idler photons reach the state with the single and joint spectra indicated by the red solid curve in Fig. 4D and the right panel in Fig. 4F, the two-photon interference fringe turns into that represented by the red dots in Fig. 4E. Now, the fringe shows a bump at around δτ = 0. The visibility *V*, obtained using the aforementioned definition, is *V* = 0.082 ± 0.019 > 0. This is as large as *V* = 0.083 ± 0.018, which is the maximum value obtained for the source when the antisymmetric component is increased by scanning the PPKTP temperature but without XPM (fig. S4B). The result demonstrates that, even throughout the XPM reshaping, the quantum entanglement of the biphoton remains, rendering the potential for application to quantum information processing.

## DISCUSSION

Reshaping over a marginal spectral distribution of single photons has been demonstrated using XPM. Lossless, low noise, and a large bandwidth nature enabled me both to reshape the spectral correlation of the photons and to tune the nonclassical interference between photons. The amount of the spectral shift (0.4 THz) was limited by the group velocity dispersion (GVD) of the PCF used. The dispersion engineering of PCFs (*45*) will be useful for reducing the GVD, which will lead to a larger modulation bandwidth (see section SV). A highly developed technology for ultrafast pulse shaping (*44*) will expand the reshaping functionalities (*25*, *33*–*36*). Lossless conversion will easily enable the simultaneous reshaping of multiple photons. These features will allow one to shape wave packets of photons for quantum networking and perform the direct manipulation of the quantum states of photons encoded in frequency, where a large computational space is naturally available. The scheme will provide an extension to the existing tools for quantum information processing that requires the coherent manipulation of the frequency property of photons.

## MATERIALS AND METHODS

### Experimental details

Output laser pulses from a mode-locked 80-MHz Ti:sapphire laser, Chameleon Ultra (Coherent Inc.) with a center frequency of approximately 756 nm and a full width at half maximum (FWHM) temporal width of 0.17 ps, were divided into two to provide the excitation pulses for SPDC in a PPKTP crystal and the control pulses for XPM. BPF1 and BPF2 were installed to obtain optimal pulse widths (*t*_0c_ and *t*_0e_ in FWHM) for each experiment. Orthogonally polarized pairs or photons were created via type II SPDC in the PPKTP crystal (*41*) and subsequently separated by a polarization beam splitter (PBS). The horizontally polarized photons (called signal photons) impinged on the PCF after combining with the co-polarized control pulse at a dichroic mirror with a signal-to-control delay Δ*T* provided by a tunable delay line installed in the optical path of the control pulses (not shown). An objective lens was used to focus the lights into the PCF, whose input facet was end-sealed. The coupling efficiencies of the signal photons and the control pulses to the PCF (measured as the transmittance including the losses of the input and output lenses) were approximately 0.7 and 0.6, respectively. The coupling efficiency of the photons to the PCF can be further improved by using a sophisticated coupling technique between correlated photons and a single-mode fiber (*49*) combined with low-loss fiber splicing between a single-mode fiber and a nonlinear PCF (*50*). The linear attenuation coefficient of the PCF, α, was <16 dB/km (catalog specification), which corresponds to a loss of less than 0.4% for a meter-long sample. The nonlinear coefficient, γ, was 11/W km at 1060 nm. The signal and idler photons passed through long-pass filters with a cutoff wavelength of 1450 nm for the control field rejection and were then coupled into single-mode fibers represented by the orange lines.

The PPKTP crystal was periodically poled along the crystallographic *x* axis with a period of 46.1 μm, yielding collinear type II phase matching between the pump (excitation pulses), signal, and idler modes around the experimental wavelengths with linear polarizations along the *y*, *y*, and *z* axes, respectively. The PPKTP crystal was housed in a temperature controller with which the crystal temperature was set at 160°C for degenerate photon pair generation.

For the reshaping experiments shown in Figs. 2 and 3, single and coincidence counts from the time-to-digital converter were recorded while scanning the center wavelengths of the TBPFs with an FWHM bandwidth of 0.4 nm inserted before the SPCMs (id210 from ID Quantique GmBH). The TBPFs were scanned with an incremental step of 0.4 nm. The SPCMs were gated synchronously with the repetition of the mode-locked laser. The quantum efficiency, dead time, and gate width of the detectors were approximately 23%, 10 μs, and 2.5 ns, respectively. Detector dark count rates were 2.6 kHz (SPCM1) and 1.6 kHz (SPCM2). As BPF1, I placed a Gaussian-shaped band-pass filter with an FWHM width of 1.19 nm and a center wavelength matched to that of the Ti:sapphire laser. No BPF2 was inserted. As a result, I obtained *t*_0c_ = 0.78 ps and *t*_0e_ = 0.17 ps (measured with an autocorrelator). The average powers of the control and excitation pulses, *P*_c_ and *P*_e_, were set at 20 and 150 mW, respectively. The experimental Δ*T* value was obtained from a comparison of Fig. 3A and Fig. 3C. All the JSI plots in this paper were obtained by subtracting the accidental coincidence counts. The pixel-dependent detection loss of photons for the data shown in Fig. 3A, which occurred as a result of the finite dead time of the SPCM1, was corrected when calculating the total coincidence count shown in Fig. 3B.

In an experiment designed to observe biphoton coalescence via HOM interference, BPF2 with an FWHM of 1.72 nm was inserted. The BPF2 reduced the spectral width of the excitation pulses and thereby the spectral width of the photon pairs (compare Fig. 2A and Fig. 4C), so that the spectral overlap between the signal and idler photons could be tuned within the bandwidth of the XPM spectral shift. Correspondingly, *t*_0c_ = 0.78 ps and *t*_0e_ = 0.60 ps. *P*_c_ = 20 mW and *P*_e_ = 4 mW (but *P*_e_ = 10 and 80 mW for single and joint spectrum measurements, respectively). Δ*T* = 0.88 and 11 ps for the data with and without XPM, respectively. The PPKTP temperature was set at 129.5°C. This setup produced the single count spectra and JSI shown in Fig. 4A and Fig. 4C (right), respectively. Then, the apparatus outlined by the dashed box in Fig. 1C was replaced with that shown in the dot-dashed box. Fiber polarization controllers were used to set the polarizations of the signal and idler photons so that they were identical at the NPBS. This was ensured by the polarizers. The normalized intensity spectral overlap was calculated as

$$\frac{\int d\omega_{s}d\omega_{i}I_{s}(\omega_{s})I_{i}(\omega_{i})}{\sqrt{\int d\omega_{s}{\left|I_{s}(\omega_{s})\right|}^{2}\int d\omega_{i}{\left|I_{i}(\omega_{i})\right|}^{2}}}$$

where *I*_s(i)_(ω_s(i)_) was the measured marginal spectra of the signal (idler) photons. The HOM dip with the application of XPM was fitted using a function

$$R(\delta \tau )=a(1-Vf(\frac{\delta \tau -\delta \tau_{0}}{w}))$$

where *f*(*x*) = 1 − |*x*| for |*x*| < 1 and *f*(*x*) = 0 elsewhere. In the fitting, *a*, *w*, δτ_0_, and *V* were free-fitting parameters, and the errors in each data point in Fig. 4B were taken into account assuming Poissonian statistics.

In the experiment that was performed to observe anticoalescence, I used band-pass filters that gave *t*_0c_ = 1.0 ps and *t*_0e_ = 0.78 ps for BPF1 and BPF2, respectively. *P*_c_ = 18 mW and *P*_e_ = 5 mW (but *P*_e_ = 10 and 80 mW for single and joint spectrum measurements, respectively). The PPKTP temperature was set at 136.0°C. A half-wave plate was placed before the PBS to blue-shift the idler photons in the PCF. This was because the red shift in our setup tends to broaden the spectral distribution (as seen in Fig. 3A) because of the dispersion of the PCF. Δ*T* (here, idler-to-control delay) = 1.1 and 11 ps for the data with and without XPM, respectively. The *V* value and the error were the mean value and the SD of 20 measurement outcomes of *V* = |(*R*_2_ − *R*_1_)/*R*_2_|, where *R*_1_ was the coincidence count at δτ = 0 and *R*_2_ was the average of coincidences at δτ = ±4 ps.

## Supplementary Material

http://advances.sciencemag.org/cgi/content/full/2/3/e1501223/DC1

## SUPPLEMENTARY MATERIALS

Supplementary material for this article is available at http://advances.sciencemag.org/cgi/content/full/2/3/e1501223/DC1 I. Numerical simulation of XPM interaction between control and signal fields II. Estimating the upper bound of HOM interference visibility from experimental JSI III. Nonlinear polarization rotation IV. Two-photon interference fringes without XPM V. Prospects for larger frequency shifts Fig. S1. Numerically calculated JSA and JSI of the photon pairs. Fig. S2. Singular values and intensity spectra. Fig. S3. Testing nonlinear polarization rotation of the signal photon wave packets induced by the control pulses. Fig. S4. Two photon interference fringes without XPM. Fig. S5. Numerically simulated evolutions of the signal field in the PCF. References (*51*–*59*)
