# Supplementary material for: Deterministic reshaping of single-photon spectra using cross-phase modulation
Source: Sci Adv. 2016 Mar 25;2(3):e1501223. doi: 10.1126/sciadv.1501223 (PMC4820381; doi:10.1126/sciadv.1501223)
Supplement: http://advances.sciencemag.org/cgi/content/full/2/3/e1501223/DC1 [file supp_2_3_e1501223__index.html]

Science Advances | Science Advances

## Supplementary Materials

**This PDF file includes:**

- I. Numerical simulation of XPM interaction between control and signal fields
- II. Estimating the upper bound of HOM interference visibility from experimental JSI
- III. Nonlinear polarization rotation
- IV. Two-photon interference fringes without XPM
- V. Prospects for larger frequency shifts
- Fig. S1. Numerically calculated JSA and JSI of the photon pairs.
- Fig. S2. Singular values and intensity spectra.
- Fig. S3. Testing nonlinear polarization rotation of the signal photon wave packets induced by the control pulses.
- Fig. S4. Two photon interference fringes without XPM.
- Fig. S5. Numerically simulated evolutions of the signal field in the PCF.
- References (*51–59*)

Download PDF

**Files in this Data Supplement:**

- Adobe PDF - 1501223\_SM.pdf
