# Supplementary material for: Deterministic reshaping of single-photon spectra using cross-phase modulation
Source: Sci Adv. 2016 Mar 25;2(3):e1501223. doi: 10.1126/sciadv.1501223 (PMC4820381; doi:10.1126/sciadv.1501223)
Supplement: http://advances.sciencemag.org/cgi/content/full/2/3/e1501223/DC1 [file 1501223_SM.pdf]

Science Advances

AAAS

advances.sciencemag.org/cgi/content/full/2/3/e1501223/DC1

Supplementary Materials for

Deterministic reshaping of single-photon spectra using cross-phase modulation

Nobuyuki Matsuda

Published 25 March 2016, *Sci. Adv.* **2**, e1501223 (2016)
DOI: 10.1126/sciadv.1501223

The PDF file includes:

I. Numerical simulation of XPM interaction between control and signal fields
II. Estimating the upper bound of HOM interference visibility from experimental JSI
III. Nonlinear polarization rotation
IV. Two-photon interference fringes without XPM
V. Prospects for larger frequency shifts
Fig. S1. Numerically calculated JSA and JSI of the photon pairs.
Fig. S2. Singular values and intensity spectra.
Fig. S3. Testing nonlinear polarization rotation of the signal photon wave packets induced by the control pulses.
Fig. S4. Two photon interference fringes without XPM.
Fig. S5. Numerically simulated evolutions of the signal field in the PCF.
References (*51–59*)

I. Numerical simulation of XPM interaction between control and signal fields

First we obtain the JSA of photon pairs generated in the PPKTP crystal. Again, the state of a signal and idler photon pair generated in a SPDC process can be written as (51)

$$|\Psi\rangle = \int d\omega_s \int d\omega_i \, S(\omega_s, \omega_i) \hat{a}_{\mathrm{s}}^{\dagger}(\omega_s) \hat{a}_{\mathrm{i}}^{\dagger}(\omega_i) \, |0\rangle \qquad (1)$$

where we neglect the vacuum and higher-order terms that will be eliminated by coincidence measurement. The JSA $S(\omega_s, \omega_i) = \alpha(\omega_s, \omega_i)\phi(\omega_s, \omega_i)$ where $\alpha(\omega_s, \omega_i)$ is the envelope function of the SPDC pump pulse and $\phi(\omega_s, \omega_i)$ is the phase matching function. In a PPKTP crystal with a length L and a poling period Λ, collinear phase matching between the pump, signal and idler modes with polarizations along the crystallographic y, y, and z axes, respectively, are satisfied when

$$\Delta k(\omega_s, \omega_i, T) = k_y(\omega_s + \omega_i, T) - k_y(\omega_s, T) - k_z(\omega_i, T) + \frac{2\pi}{\Lambda} = 0 \qquad (2)$$

where $k_{y(z)}(\omega, T) = n_{y(z)}(\omega, T)\omega/c$ is the wavenumber along the propagation axis, $n_{y(z)}(\omega, T)$ is the refractive index of the material for the light with $y(z)$ polarization and T is the crystal temperature. $\phi(\omega_s, \omega_i) = \mathrm{sinc}(\Delta k(\omega_s, \omega_i, T)L/2)$, where the unimportant global phase is omitted.

In Fig. S1 we plot numerically calculated JSA = $S(\omega_s, \omega_i)$ (A) and JSI = $|S(\omega_s, \omega_i)|^2$ (B) as a function of the signal and idler wavelengths λ_s and λ_i. For the material dispersion we used Sellmeier equations in (52, 53) with the temperature dependence in (54). The temperature expansion coefficient of KTP (54) was also taken into account for Λ. Since these equations are empirical, it was also necessary to additionally multiply with a coefficient of 2.8 for the temperature dependence to reproduce the wavelength of the photon pairs obtained in the experiment.

JSA is expressed by a complete set of orthonormal basis functions that can be obtained by the Schmidt decomposition as follows (55).

$$S(\omega_s, \omega_i) = \sum_j \sqrt{\lambda_j}\, A_{s,j}(\omega_s) A_{i,j}(\omega_i) \tag{3}$$

where $A_{s,j}(\omega_s)$ and $A_{i,j}(\omega_i)$ are orthonormal functions of the signal and idler wavefunctions, and λ_j are non-negative, real numbers satisfying $\Sigma_j \lambda_j = 1$. The basis functions and the coefficients can be numerically obtained using singular value decomposition (SVD) (51).

In the measurement of the delay-dependent marginal frequency distribution of heralded signal photons (shown in Fig. 3(A)), a bandpass filter (TBPF2) is applied to the idler photons before detection. The effective Schmidt mode of the biphoton state after the filtering operation can be approximated by decomposing the JSA multiplied by the applied filter function (56):

$$S'(\omega_s, \omega_i) = T(\omega_i) S(\omega_s, \omega_i) = \sum_j \sqrt{\lambda'_j}\, A'_{s,j}(\omega_s) A'_{i,j}(\omega_i) \tag{4}$$

where $T_i(\omega_i)$ is the transmission function of TBPF2, which is Lorentzian-shaped with a center wavelength of 1512 nm (almost tuned to the center wavelength of the marginal idler spectrum) and a FWHM of 0.4 nm in intensity.

Computed singular values λ_j and λ'_j are plotted in fig. S2(A). We see that the mode with the largest singular value dominates the JSA with TBPF2. For the mode, the Schmidt basis function of the signal field without and with the TBPF2 resemble each other as shown in fig. S2(B). Therefore, the heralding detection with TBPF2 will eventually filter out the first Schmidt mode of the signal photon $A_{s,1}(\omega_s)$. Based on this approximation, we now only consider the first term $A_{s,1}(\omega_s)\, A_{i,1}(\omega_i)$ as the initial JSA before the XPM interaction.

Next we numerically simulate the nonlinear pulse evolution between the pump and signal fields. For the latter $A_{s,1}(\omega_s)$ is used based on the above approximation where the initial JSA is

separable. The nonlinear evolution is governed by the following coupled nonlinear Schrödinger equations (NLSEs) (25).

$$\frac{\partial A_c}{\partial z} + \frac{\alpha}{2} A_c + \sum_{k\geq 2} \frac{i^{k+1}}{k!} \beta_{c,k} \frac{\partial^k A_c}{\partial {t_c}^k} = i\gamma |A_c|^2 A_c \quad (5)$$

$$\frac{\partial A_s}{\partial z} + \frac{\alpha}{2} A_s + d\frac{\partial A_s}{\partial t_c} + \sum_{k\geq 2} \frac{i^{k+1}}{k!} \beta_{s,k} \frac{\partial^k A_s}{\partial {t_c}^k} = i2b\gamma |A_c|^2 A_s \quad (6)$$

Here $A_{c(s)}$ represents the slowly-varying envelope and $\beta_{c(s),k}$ is the k-th order propagation constant at the control and signal frequencies. We use $A_{s,1}$ for A_s. Because the theory is linear in the signal field operators, single photon fields obey the same linear equations of motion as do weak classical fields (21) (see Ref. (57) for a numerical simulation for the entire frequency-entangled JSA). α is the linear attenuation coefficient, which is assumed to be wavelength independent (− 0.016 dB/m from catalog). XPM induced by the signal photon wavepackets is neglected. $t_c = t - z\,\beta_{c,1}$ is the time measured in a reference frame of the control pulses. $d = \beta_{s,1} - \beta_{c,1}$ is the differential group delay between the control pules and signal photons. γ is the nonlinear constant. The catalogue value of γ is 0.011 /W/m, which is used for the value at around the control wavelength. b ($0 \leq b \leq 1$) is the parameter associated with the degraded spatial overlap between mode fields with the greatly separated wavelengths (756 nm and 1512 nm) (58). Terms associated with four-wave mixing, which is not phase matched, are ignored. A symmetric split-step Fourier method was employed for the numerical calculation of the coupled NLSE with up to 6-th order propagation constants (extracted from Fig. 1(B)) taken into account. In the calculation b and d are the only free parameters.

Fig. 3(C) in the main text shows the numerical result obtained in accordance with the above procedure, along with the parameters b and d of 0.6 ps/m and 0.7, respectively. The plot well captures the behavior of the experimental result (Fig. 3(A)), and includes the major blue and red shifts found at the positive and negative delays, respectively. The d value for the fitting is in a reasonably small range considering the wavelength-dependent variation of the group velocity (Fig. 1(B)). The obtained b value was less than unity. This is mainly due to the wavelength dependence of the effective modal area in the PCF, which reduces the lateral field overlap (58).

II. Estimating the upper bound of HOM interference visibility from experimental JSI

In the HOM interference experiment, the coincidence count rate after the photons have passed through the NPBS can be described as follows (59):

$$R \propto \int d\omega_s \int d\omega_i \left| S(\omega_s, \omega_i) e^{i(\omega_s t_s + \omega_i t_i)} - S(\omega_i, \omega_s) e^{i(\omega_i t_s + \omega_s t_i)} \right|^2 \qquad (7)$$

where t_s and t_i are the detection timings of the signal and idler photons. Accordingly, for a known $S(\omega_s, \omega_i)$ we can obtain a two-photon interference fringe by plotting R as a function of the arrival time difference between photons $\delta t = t_i - t_s$. However, we cannot reconstruct the phase information of JSA from the experimental JSI. Nonetheless, we can obtain the lower bound visibility of the two photon interference fringe from $|\mathrm{JSA}| = \sqrt{\mathrm{JSI}}$; any complex phase terms in JSA will induce an increase in R and thus decrease in the visibility (59). Substituting $S(\omega_s, \omega_i)$ in Eq. (7) with the square root of the experimental JSI after XPM reshaping (Fig. 4(C), right panel), we obtain dip visibility to be 91%. The value well describes the experimental value (87 ± 1)% without accidental coincidence counts. The imperfection of the estimated visibility mainly originates from the asymmetric component of JSI with respect to the zero-detuning axis $\omega_s = \omega_i$, which results in the distinguishability in the single count spectra.

III. Nonlinear polarization rotation

The two photon interference fringes shown in Fig. 4(B) and (E) exhibited a reduction in coincidence counts when the photons interacted with the control pulses. This is due to the nonlinear polarization rotation (25) of the photons induced by the control pulses originating from the fact that the PCF that was used was slightly birefringent. This is confirmed as follows. First, using the setup shown in fig. S3(A), we recorded the coincidence count as a function of signal-to-control delay ΔT for the cases with and without the polarizer (PL) in the signal channel in front of the SPCM. These results are shown in fig. S3(B) as closed and open blue dots. It is

clear that the coincidence loss occurred when PL was used. This indicates the polarization rotation of the signal photons, which was also detectable in the two-photon interference setup with polarizers in place. For comparison we also plot coincidence counts $R_{\mathrm{classical}}$ obtained for the experiment shown in Fig. 4(B) as a function of ΔT (red closed squares). The behavior is explained by the closed blue dots. This polarization rotation can be simply eliminated by matching the birefringent axis of the PCF with the polarization axis of the control and signal fields, as in previously reported experiments such as (30).

IV. Two-photon interference fringes without XPM

Figure S4(A) shows a two-photon interference fringe with maximum visibility observed without XPM in the set-up for biphoton distinguishability reduction. The experimental conditions are as follows: PPKTP crystal temperature: 162°C, BPF2 bandwidth: 1.72 nm (temporal width: 0.60 ps), laser average power for PPKTP excitation: 3 mW. The visibility of the dip is obtained by fitting (indicated by the solid line) as 91.7 ± 2.2 %. Figure S4(B) shows the two-photon interference with the maximum bump observed without XPM with the set-up for frequency entanglement detection. The experimental conditions are as follows: PPKTP crystal temperature: 120°C, BPF2 bandwidth: 1.19 nm (temporal width: 0.78 ps), laser average power for PPKTP excitation: 4 mW. The bump visibility (height) is described in the method section.

V. Prospects for larger frequency shifts

We explore how the finite fiber dispersion affects the frequency shifts using the numerical simulation introduced in Sec. I. In fig. S5(A), we plot the evolution of the signal pulse wavepacket $A_{s,1}$ under the experimental conditions described in Sec. I. Then, we set d = 0 ps/m, which can be realized by tuning the wavelengths of the signal and control fields. Also, by optimizing the temporal width of the Gaussian-shaped control pulses (t_{0c} = 2.5 ps), we obtained the result shown in fig. S5(B). The data indicate that a frequency shift larger than the bandwidth of the signal field is available. Furthermore, we plot the results obtained when both the group velocity dispersions $\beta_{c,2}$ and $\beta_{s,2}$ were 50% and 20% of those of the PCF used in figs. S5(C) and

(D), respectively. Hence, the reduced GVD leads to larger spectral shifts. We could also further tailor these results by modifying the temporal shape of the control pulses.

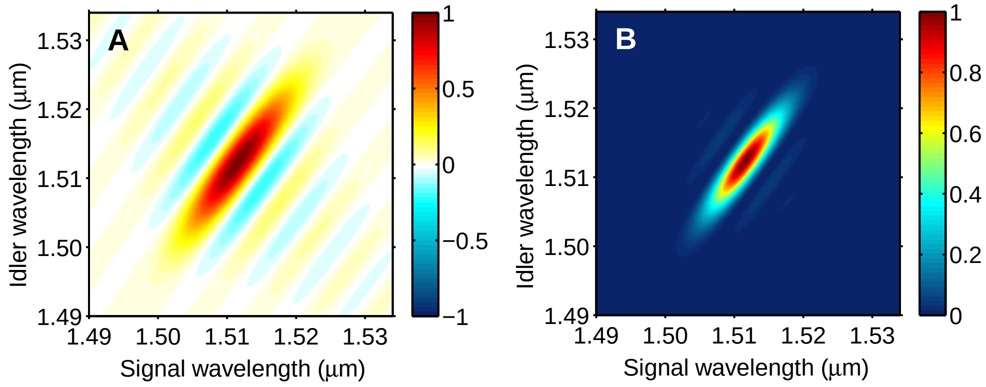

fig. S1 Numerically calculated JSA (real part) (A) and JSI (B) of the photon pairs generated via SPDC in a PPKTP crystal using the experimental parameters.

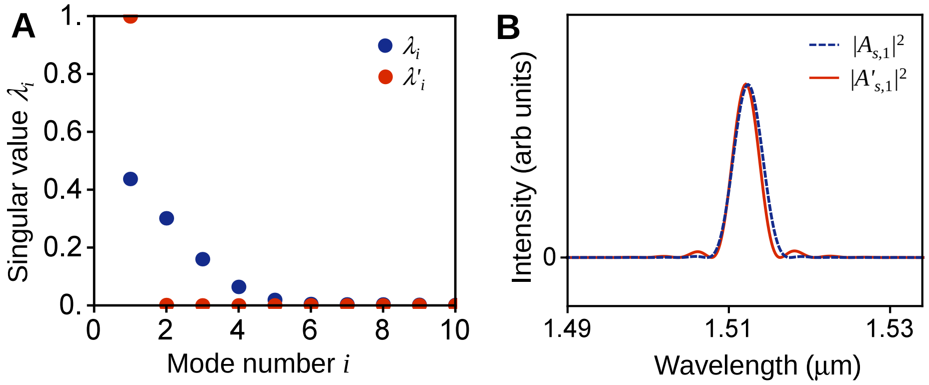

fig. S2 (A) Singular values and (B) intensity spectra of the Schmidt mode of the signal photons. These are obtained from the Schmidt decomposition of JSA with and without TBPF2 in the idler channel. Note that both $A_{s,1}$ and $A'_{s,1}$ are real.

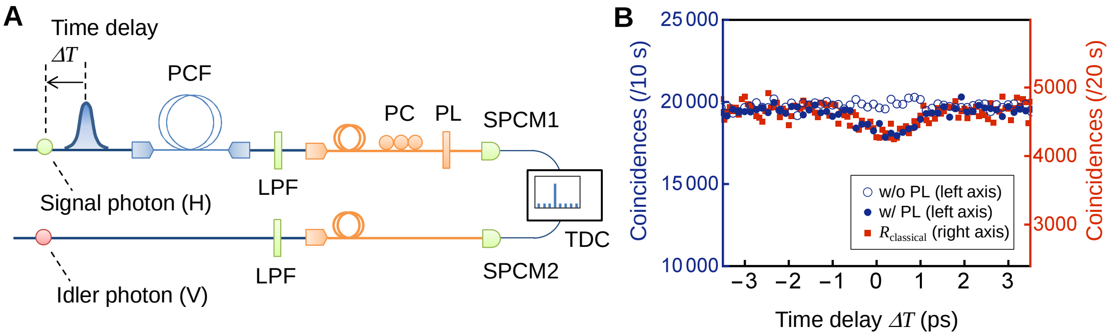

fig. S3 Testing nonlinear polarization rotation of the signal photon wave packets induced by the control pulses. (A) Experimental set-up. See the caption of Fig. 1(C) for abbreviations. (B) Coincidence count versus signal-to-control time delay ΔT without and with the polarizer (PL) in Fig. S3(A). For comparison, $R_{\text{classical}}$ for the HOM experiment with the control pulses shown in Fig. 4(B) is overlaid. The detection loss of photons due to the dead time of the SPCMs were corrected.

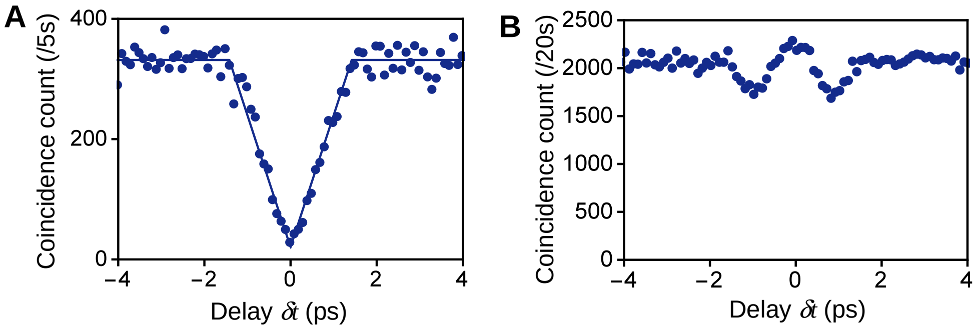

fig. S4 Two photon interference fringes that exhibits (A) maximum dip and (B) maximum bump without XPM.

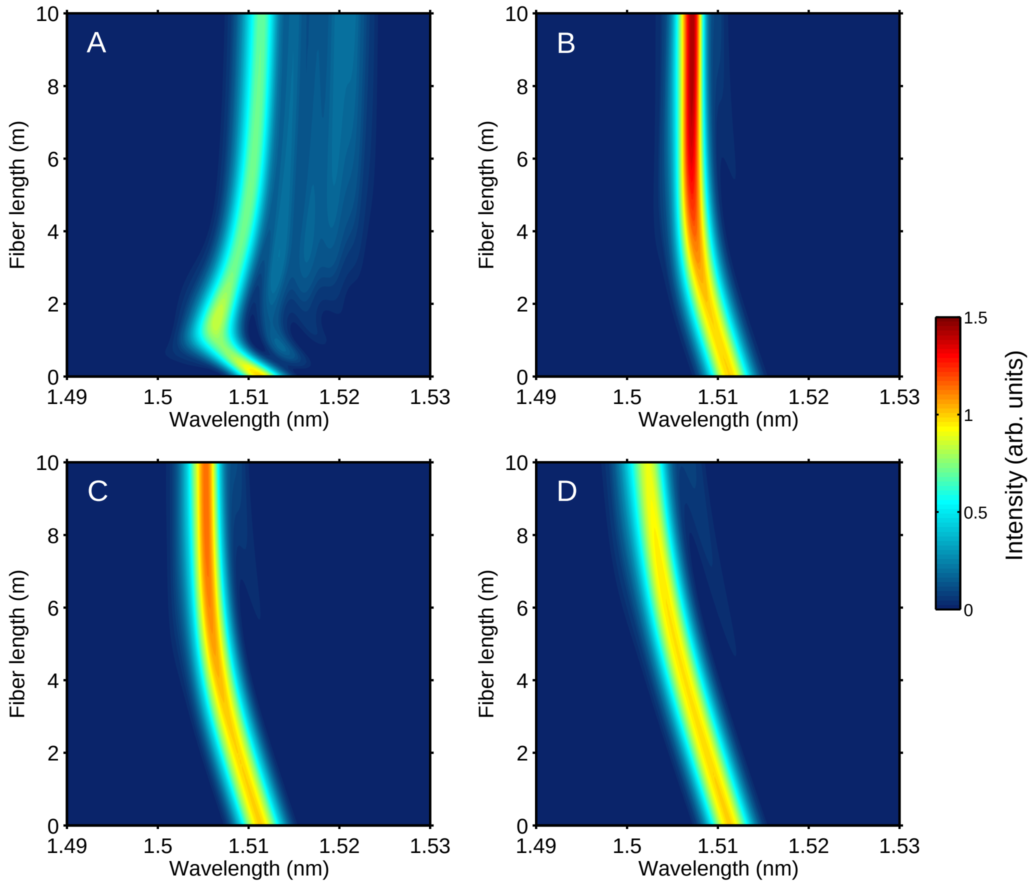

fig. S5 Numerically simulated evolutions of the signal field in the PCF. (A) With the conditions described in Supplementary Sec. I (d = 0.6 ps, the signal-to-pump delay $\Delta T = -0.9$ ps, the temporal width of the control pulse t_{0c} = 0.78 ps, $\beta_{c,2}$ = 36 ps^2/km, $\beta_{s,2} = -60$ ps^2/km). (B) A result obtained by setting d = 0 and t_{0c} = 2.5 ps at $\Delta T = -1.2$ ps. The other parameters are unchanged from (A). (C), (D) Results when both $\beta_{c,2}$ and $\beta_{s,2}$ are 50% (C) and 20% (D) of those of the PCF. All the plots show spectral intensities and share the same color bar.
